# Nano-Based Advanced Thermoelectric Design: Materials Engineering and Thermal Management Applications

**DOI:** 10.3390/nano16201277

**Published:** 2026-10-09

**Authors:** Jin Wang

**Affiliations:** School of Energy and Environmental Engineering, Hebei University of Technology, Tianjin 300401, China; wjwcn00@163.com

The global pursuit of sustainable energy solutions and efficient thermal management has propelled thermoelectric (TE) technology to the forefront of materials science research. Thermoelectric devices, capable of directly converting temperature gradients into electrical energy and vice versa, offer unique advantages including no moving parts, compact form factors, and precise thermal control. However, the widespread adoption of TE technology has long been constrained by relatively low conversion efficiency, traditionally quantified by the dimensionless figure of merit. The intricate coupling among the Seebeck coefficient, electrical conductivity, and thermal conductivity presents a fundamental challenge: optimizing one parameter often degrades another. This Special Issue, “Nano-Based Advanced Thermoelectric Design”, brings together four original research articles and one comprehensive review that collectively address this challenge through innovative nanoengineering strategies, spanning fundamental material design to practical thermal management applications.

The nanoscale dimension offers unprecedented opportunities to decouple these traditionally interdependent transport properties. As reviewed by Latronico et al. [1], one-dimensional (1D) thermoelectric nanomaterials, i.e., nanowires and nanotubes, exhibit quantum confinement effects that fundamentally alter their electronic and phononic transport behaviors. The review systematically examines how reducing material dimensions can circumvent the primary limitations of bulk thermoelectric materials, with particular emphasis on silicon nanowires and carbon nanotubes. Notably, carbon nanotubes offer additional advantages of mechanical flexibility and earth-abundant source materials, making them promising candidates for flexible thermoelectric generators. The authors also highlight the emerging role of machine learning techniques in predicting electron relaxation times and lattice thermal conductivity, signaling a paradigm shift toward theory-guided experimental design in thermoelectric research.

Building on the foundation of nanoscale transport engineering, Dores et al. [2] present a compelling experimental demonstration of enhancing thermoelectric performance in bismuth telluride (Bi_2_Te_3_) and antimony telluride (Sb_2_Te_3_) thin films through thermal co-evaporation with their constituent elements. A key insight from this work is that enriching commercial alloys with their own constituent elements, rather than introducing foreign dopants, effectively enhances thermoelectric properties. The successful fabrication of a ring-shaped thermoelectric device with six p-n junctions, achieving a temperature differential of 7.1 °C at 4.8 mA, further validates the practical potential of these optimized films for localized heating and cooling applications.

The integration of thermoelectric materials with phase change materials (PCMs) represents another innovative pathway for advanced thermal energy management, as demonstrated by Jie et al. [3]. Their work addresses the inherent limitations of organic PCMs—melting-induced leakage, poor solar absorption, and low thermal conductivity—through a hybrid filler strategy employing expanded graphite (EG) and carbon nanotubes (CNTs). The worm-like porous structure of EG provides capillary confinement to prevent leakage while forming three-dimensional thermal conduction networks after compression molding. The double-layer composite design, featuring a CNT-rich top layer for efficient solar harvesting and a thermally conductive bulk layer for latent heat storage, achieves a remarkable solar absorptance of 92.96%.

While the aforementioned studies focus on optimizing thermoelectric materials and systems, the practical deployment of thermoelectric technology also requires careful consideration of thermal comfort in real-world applications. Wang et al. [4] contribute an experimental study on radiative cooling vests designed to improve human thermal comfort in high-temperature outdoor environments. Although not directly addressing thermoelectric materials, this work exemplifies the broader context of personal thermal management, where thermoelectric cooling plays a complementary role. The study systematically evaluates the effects of coating thickness on radiative cooling performance, identifying an optimal thickness of 160 μm that reduces the inner surface temperature of the vest by up to 5.54 °C compared to standard vests. This work underscores the importance of human-centric evaluation in thermal management technologies, and highlights the potential for integrating thermoelectric cooling with passive radiative cooling strategies for enhanced personal thermal comfort.

From a fundamental perspective, Fang et al. [5] employ the lattice Boltzmann method (LBM) to investigate micro-lubrication and heat transfer in wedge-shaped channels with micro-convex surface textures. This study addresses tribological phenomena, providing valuable insights into microscale heat transfer mechanisms that are relevant to thermal management systems. The authors found that microconvex textures increase the maximum oil film pressure by approximately 4.34%, which provides a basis for understanding how surface engineering at the microscale affects heat transfer. This work exemplifies the type of fundamental transport modeling for the rational design of thermal management systems involving thermoelectric elements.

These contributions illustrate the multidimensional nature of advanced thermoelectric design, from the quantum confinement effects in 1D nanomaterials to the practical optimization of thin-film deposition parameters, from the integration of thermoelectric generators with phase change materials to the human-centric evaluation of cooling garments, and from fundamental microscale transport modeling to machine learning-assisted materials discovery. The common thread across these diverse studies is the recognition that nanoscale engineering provides powerful tools to manipulate thermal and electrical transport properties. As the thermoelectric community continues to push device performance, the integration of computational prediction, experimental synthesis, and application-driven design will be essential for translating laboratory breakthroughs into commercially viable technologies. We hope that this Special Issue will inspire further cross-disciplinary collaborations and accelerate the development of next-generation thermoelectric materials and devices for sustainable energy harvesting and precise thermal management.
